# Hospital Medical and Nursing Managers’ Perspective on the Mental Stressors of Employees

**DOI:** 10.3390/ijerph17145041

**Published:** 2020-07-13

**Authors:** Britta Worringer, Melanie Genrich, Andreas Müller, Harald Gündel, Contributors of the SEEGEN Consortium, Peter Angerer

**Affiliations:** 1 Institute for Occupational, Social, and Environmental Medicine, Centre for Health and Society, Medical Faculty, Düsseldorf University, 40225 Düsseldorf, Germany; peter.angerer@uni-duesseldorf.de; 2 Institute of Psychology, Work & Organizational Psychology, University of Duisburg-Essen, 45141 Essen, Germany; melanie.genrich@uni-due.de (M.G.); andreas_mueller@uni-due.de (A.M.); 3 Department of Psychosomatic Medicine and Psychotherapy, Ulm University Medical Center, 89081 Ulm, Germany; Harald.Guendel@uniklinik-ulm.de

**Keywords:** psychosocial stress, occupational health, healthcare, leadership, employee mental well-being, qualitative research, psychosocial safety climate

## Abstract

Working conditions in hospitals are characterized by occupational stressors, which lead to potentially harmful psychosocial stress reactions for medical and nursing staff. Representative surveys showed that almost every second hospital physician or nurse is affected by burnout and that there is a strong association between leadership behavior and employee health. Workplace health promotion programs can only be successful and sustainable if managers support them. However, it is still unclear whether hospital managers are aware of the working conditions and perceive them as an influence on the health of their employees. Therefore, the aim of this qualitative study was to explore the hospital medical and nursing managers’ perspective on the mental stress of their employees. Semi-standardized interviews with 37 chief physicians (CP), senior physicians (SP) and senior nurses (SN) in total were carried out in one German hospital. The interviews were content-analyzed based on the guideline for the mental risk assessment of the ‘Gemeinsame Deutsche Arbeitsschutzstrategie’ (GDA). Most reported work characteristics related to work organization, work task, and social factors. Staff shortage could be identified as an underlying stressor for several other burdens. Social support by managers and among colleagues was mentioned as main resource. The findings indicate that managers strive to reduce the burden on their staff, especially through their personal support. Nevertheless, it seemed that managers need additional resources to counteract stressors.

## 1. Introduction

Working conditions hospitals comprise of several occupational psychosocial stressors, which lead to potential stress reactions and health problems for medical and nursing staff [1,2,3]. The Sixth European Working Conditions Survey 2020 showed that compared to other professions, workers in the health care sector experience the greatest work intensity, the most frequent interruptions, high emotional demands because of increasing number of aggressive patients or relatives, and that there is the highest percentage of workers subjected to social stressors, for example bullying, humiliating behavior or physical violence [4]. The Fourth European Working Conditions Survey reported that 40 percent of employees in the healthcare sector suffer from constant health problems [5]. Other representative surveys showed that almost every second (48.7%) surgical hospital physician in Germany [6] and almost one in three nurses [7] is affected by burnout.

In addition to the health effects on employees, impaired mental health of employees due to poor working conditions can lead to an increased risk of presentism, sick leaves, decreased performance [8], medical errors [9], and intentions to lay off are further negative effects [10,11], which can lead to a potential economic harm [12]. A study by Han, Shanafelt [13] could illustrate this financial effect with regard to hospitals in the USA: this study calculated that on average the annual economic cost associated with burnout of physicians is approximately $7600 per employed physician due to physicians’ turnover or reduced clinical working hours.

The specific working conditions, which are associated with an increased risk of illness have been scientifically investigated. Rau and Buyken [14] evaluated 54 publications and found that high work intensity, low decision latitude, job strain, effort–reward imbalance, overtime hours, long working hours, certain forms of shift work, low social support, role stress, bullying, and job insecurity should be considered as potentially hazardous to health and should therefore be considered in risk assessments. More specifically, in two comprehensive systematic reviews, Theorell, Hammarström [15] found that high psychological demands and low decision latitude, and low decision latitude and bullying have a significant impact on the development of depressive symptoms. Likewise, Theorell, Jood [16] could demonstrate that low decision latitude, job strain, or noise have an increased incidence of ischemic heart disease.

On the basis of these scientific findings, lists have been drawn up by the International Labour Organization (ILO) for the most important characteristics of occupational stress and psychosocial working conditions and, consequently, proposals for the assessment and design of work [17]. In the national regulations in Germany, these are presented in the so-called Joint German Occupational Safety and Health Strategy (GDA) guidelines, which the companies should follow [18]. The GDA is a permanent platform set up by the federal government, the federal states and accident insurance providers in the Occupational Health and Safety Act and Social Code Book (SGB) VII. Originally resulting from European and international obligations, the GDA has now become firmly established in the German occupational safety system. The aim of the alliance is to continuously modernize the occupational health and safety system in Germany in line with the changing world of work and to create incentives for companies to further strengthen the safety and health of employees. The guideline for mental risk assessment of the GDA can be used to check whether the characteristics that are essential for the respective company from the subject areas ‘work task’, ‘work organization’, ‘recognizable social factors’, ‘work environment’, and ‘work equipment’ have been taken into account.

Managers play a key role in risk assessment and the resulting improvement in psychosocial working conditions. It has been shown that support from managers is one of the key factors for the success or failure of organizational health promotion interventions [19,20]. Lacking support from managers has been revealed as one of the main problems for the successful implementation of health promoting measures [21,22]. Specifically, managers must allocate time and human resources in order to make measures feasible [23,24]. Furthermore, managers need to inform their employees about the interventions, should communicate the intervention objectives in a motivating way, and enable their employees to participate [25,26]. Eventually, it is the managers who decide whether a health-promoting measure is implemented at all. However, earlier research indicates that managers are not fully understanding the psychosocial resources and stressors of their employees [27].

It is unclear whether hospital managers are aware of the working conditions relevant from a scientific and legal point of view and perceive them as an influence on the health of their employees, which we assume would be a prerequisite for their willingness to improve working conditions. For this reason, the study aims to investigate which of the known stressors and resources are perceived by hospital senior managers in their area of responsibility, what other psychosocial factors are considered as significant, and what importance they attribute to them.

## 2. Materials and Methods

### 2.1. Study Design

To investigate medical and nursing managers’ perspective on the mental stress of their employees, we conducted a qualitative interview study. We conducted semi-standardized individual interviews with medical and nursing managers of one German hospital with two locations, belonging to a commercial hospital company. The bigger one has about 500 beds and employs about 700 physicians and nursing staff. The smaller one has around 350 beds and about 450 medical and nursing employees, and was converted from a specialized clinic to an acute care facility. Our interview study was approved by the ethics committee of the University of Düsseldorf (Registration-ID: 2017114495). We also informed the clinic’s works council and data protection authorities about the content and progress of the study. All approvals were available before the interviews were conducted.

The interviews were content-analyzed based on the guideline for the mental risk assessment of the GDA [18] with the category system (see Appendix A Table A1) consisting of the five main categories: ‘work task’ (workloads resulting from the task itself), ‘work organization’ (workloads resulting from organizational processes of work), ‘recognizable social factors’ (workloads caused by social relationships at work), ‘work environment and work equipment’ (workloads caused by physical and chemical factors, workplace design and equipment), and ‘new working conditions’ (workloads that arise, for example, through spatial mobility, time flexibility, or reduced separation between work and private life). Each main category was subdivided into further subcategories, based on acknowledged work stress and work design models, such as the effort–reward imbalance model [28], the job demand resources model [29], or the model of organizational justice [30].

We have distinguished between positive (resources) and negative (stressors) connoted statements. We defined quotes as a resource when managers perceived the work characteristic as psychologically health-strengthening, and as a stressor when managers perceived the mentioned work characteristic as psychologically harmful. For example, decision latitude, which by definition is a resource, can lead to overstrain if the level is too high see, Kubicek, Korunka [31].

### 2.2. Recruitment

Interviews were performed from April 2018 to July 2018. Participation was voluntary but recommended by the hospital’s managing director, medical directors, and nursing service management. The participants were allowed to conduct the interviews during their working hours. The recruitment of chief physicians (CP), senior physicians (SP), and senior nurses (SN) was done by (1) informing about the interview study at conferences of CP and SN, (2) calling attention to the study by e-mailing participants study information, and by (3) coordinating appointments by telephone to further inform about the study, partly via secretarial offices. The interview dates were arranged personally by telephone or e-mail with the participants and carried out ‘Face-to-Face’ on site at the clinic in the office of the manager after informing about the data protection regulations and signing consent declaration. The interviews were conducted by a certified pedagogue with systemic qualification, a psychologist and psychotherapist, and a medical student with experience as an examined nurse. The interview and the interview guide were tested in advance through trial interviews. It was almost always possible to conduct the interviews without interruptions (e.g., through emergency treatment). Upon approval by the managers, the conversations were recorded and lasted 45 min on average.

### 2.3. Design of Interview

We used an interview guideline as a basis for the interviews. We asked the managers about their perception of the most important organizational stressors and resources for their employees (‘What do you think are the most important work stressors for your employees?’ and ‘Which working conditions do you consider supportive and motivating for your employees?’). Further questions were asked regarding the managers’ attitude towards health-related job measures, perceived organizational norm and perceived behavioral control, which are not the focus of this study. However, statements that addressed the participants’ perspective on mental stress or resources of employees were included in this analysis. These questions were: ‘To what extent do you see a connection between the stressors you have just mentioned and the mental health of your employees? How do you describe your own role as a supervisor in relation to the mental health of your employees at work?’, ‘How important is the mental health of employees in your hospital? What opinions do your colleagues, have on the subject?’, and ‘What changes do you think can be implemented to relieve the strain on your employees in their day-to-day work? Do you personally see opportunities for yourself to maintain the ‘mental’ health of your employees at work and to reduce the stress you mentioned?’.

The draft interview guide was discussed by the study team, tested in an expert interview with a physician from one university hospital and then slightly modified. The interviews were conducted by three interviewers of the study team. After conduction of the first six interviews, there was another discussion to find out if it was necessary to make modifications. No changes were necessary. The interviews were conducted until “theoretical saturation” was achieved. Glaser and Strauss [32] defined these points of analysis at which “no additional data are being found whereby the (researcher) can develop properties of the category. As he sees similar instances over and over again, the researcher becomes empirically confident that a category is saturated. When one category is saturated, nothing remains but to go on to new groups for data on other categories, and attempt to saturate these categories also” [32,33] (p.61).

### 2.4. Data Analysis 

The transcription of the digitally recorded interviews was acquired by a specialized company and then analyzed by the study team using the conventional structuring content analysis [34,35,36] and by using the digital software MAXQDA 2018.1. We used the structuring content analysis, which pursues the goal of summarizing and systematizing the contents of the interviewees by theoretically derived dimensions in such a way that the results can be understood intersubjectively [37,38]. The creation and application of the category system was based on the deductive process using the main and subcategories of the GDA guideline for mental risk assessment. Definitions, anchor examples and coding rules were defined for these upper and subcategories. We tested the reliability in two steps: At first, the formative reliability test, three members of the study team applied the coding guidelines based on three selected transcriptions and then checked and discussed the results to similarities and differences. The results were discussed with the large study team including the project leaders. Minor modifications were made to the coding guide. In the second step we tested the summative reliability. For this purpose, 2 × 4 transcribed interviews were coded by one researcher and counter-coded independently by another researcher. Based on the results, the inter-rater reliability was calculated. A Cohen’s kappa value of 0.82 indicated good reliability of our coding system [39]. In the first review of the transcriptions, the statements of the managers were assigned to the categories created. In the next step, we analyzed the statements within a category for a possible more in-depth systematization. We counted the number of mentioned work characteristics by separating them into stressors and resources per main and subcategory as follows: each named stressor or resource per subcategory and interviewee was counted once. Double mentions within an interview were counted once.

### 2.5. Sample

We interviewed 37 managers in total, including 14 CP, 9 SP, and 14 SN working in different medical departments. We received no information from 6 SNs about the duration of employment in the current clinic, and 1 CP and 5 SNs lacked information on age. From the information available to us, the age range was from 34 to 60 years with a mean age of 51.9 years (CPs), 43.6 (SPs), and 47.9 (SNs). The average time of being employed in the current clinic was 5.4 years for CPs, 8.7 years for the SPs, and 23.4 years for SNs (Table 1). 

## 3. Results

**Figure 1:**
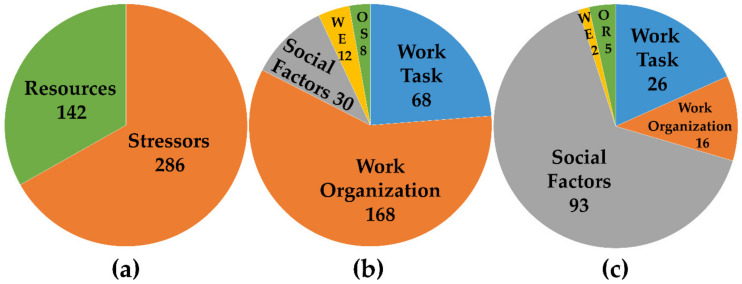
Graphical representation based on Table A2 and Table A3 of the number of mentioned resources or stressors across all occupational groups (**a**), the number of mentioned stressors per work characteristic across all occupational groups (**b**), and the number of mentioned resources per work characteristic across all occupational groups (**c**). WE = Work Environment; OS = Other Stressors; OR = Other Resources.

By counting the number of mentioned work characteristics per manager and subcategory, we perceived that managers reported more stressors (Table A2) compared to resources (Table A3; Figure 1a). With regard to the reported frequencies of the characteristics, we found no qualitative difference in the weighting of the stressors or resources regarding the main categories between the different occupational groups (CP and SP versus SN), nor did we find differences between the hierarchical levels (CP versus SP; Table A2 and Table A3).

Most frequently reported stressors were related to the work organization within all groups, followed by stressors of the work task. Stressors due to social factors or due to unfavorable working environment were less frequently named (Figure 1b).

Again, regarding reported frequencies of the characteristics we found no qualitative difference in the weighting of the resources regarding the main categories between the different occupational groups (CP and SP versus SN), nor did we find differences between the hierarchical levels (SP versus CP). Resources related to social factors were consistently reported most frequently by all groups, followed by resources from the work task. Resources due to work organization or due to unfavorable working environment were less frequently named (Figure 1c).

**Figure 2:**
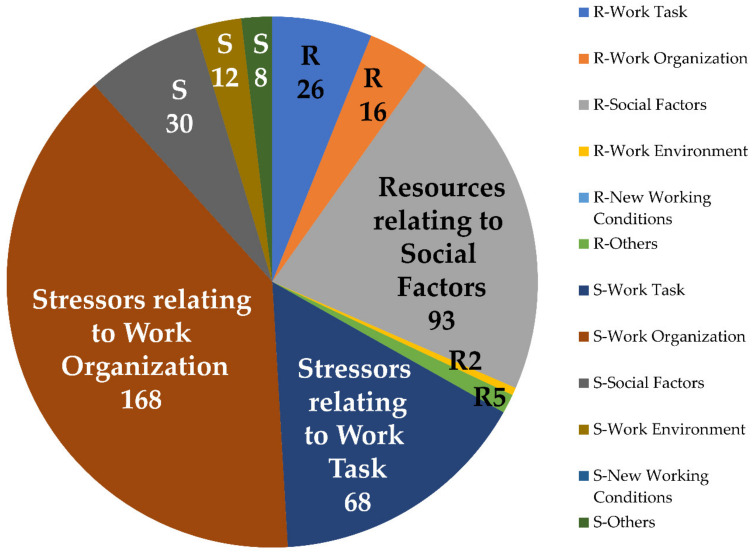
Graphical representation of the distributions based on the number of topics mentioned per manager and subcategory of both resources and stressors. The distribution is based on the count in Table A2 and Table A3. R = Resources; S = Stressors.

Altogether, stressors relating to work organization were by far the most frequently named, followed by resources relating to social factors, and stressors relating to the work task (Figure 2).

### 3.1. Managers’ Perspective on the Influence of Work Task on the Employees

In general, regarding reported frequencies of the characteristics both stressors and resources due to the work task were mentioned second most by managers. Within the category work tasks, stressors relating to emotional demands were most frequently reported, followed by stressors relating to responsibility, decision latitude, and qualification. Rather few stressors were reported due to the completeness of the task, the information offered, or the variability of the tasks (Table A2, Figure 1b). Regarding resources relating to the work task, resources regarding decision latitude were most frequently reported, followed by resources relating to emotional demands, qualification, information offer, and variability. Resources due to the completeness of the task or responsibility were not mentioned (Table A3, Figure 1c).

#### 3.1.1. Emotional Demands

With respect to the work characteristic emotional demand we found an ambiguity in the statements. Regarding stressors related to emotional demands of the work task, four SP, six CP, and five SN reported that they observed employees’ stress due to emotional demands of the work task. However, a large positive part of the hospital work seems to be nourished by experiencing good courses of treatment and satisfied patients (one CO, two SP, and four SN).

‘When we had or have a critical patient on the ward and we see this success, that we have mobilized him regularly, that we have taken time for him, this, there is no need to say much, no staff, little time. But because we still wrested the time from somewhere, we mobilized the patient regularly. And we simply see that the patient is on the road to recovery. And that is just, it was worth it’.

(SN 24)

At the same time, dealing with serious illness and the death of patients and grief of their relatives, especially when dealing with severely ill young patients or when watching an acute life-threatening event, is one of the greatest emotional burdens (two CP, one SP, and five SN).

‘I have seen a colleague coming out of the patient’s room, a young patient died, died alone, that is the worst, and she came out crying’.

(SN 16)

‘This can sometimes be a traumatic experience with a dying patient, the first death certificate, the first post-mortem that you have to do’.

(CP 9)

Conflicts between patients and their relatives (two CP), especially in the case of aggressive patients (two CP), or when the staff is personally threatened (one CP), are further factors that can cause emotional stress.

‘In other words, to the point of employees being personally threatened and not always daring to go into the underground car park when they go home at 9 p.m.’

(CP 9)

On the other hand, we identified burdens, which are also due to the factor staff shortage. Seven managers mentioned stressors as a result of staff shortage and thus lack of time such as that their employees have to endure patients’ suffering (three SN and one SP), because they cannot be up to the task (one CP and two SN).

‘So, because you know of course that you could do it better, but I only have two hands and I can’t help it. I have to leave one of them suffering’.

(SP 35)

Another cluster of stressors mentioned was when employees have to turn off life support devices (one CP), to remove organs from dying patients (one CP), or when they have to make treatment decisions for seriously ill patients (one CP).

‘Explantations, i.e., the removal of organs from patients, are certainly such a stressful situation, … and when you’re about to turn off some equipment, it’s things like that’.

(CP 2)

Managers also mentioned, that they perceived stress among their employees, because they had to bring bad news to patients or relatives and had to have incriminating conversations with relatives (two CP and one SP). One SP and two SN reported stress of the employees due to emotional closeness to the patient, which sometimes leads to worrying about the state of health of a patient even after work. One SP also stated to see difficulties for their employees, because they did not get positive feedback or positive appreciation about the course of a disease due to the job rotation system. Another SP said that there was a continuing basic tension due to the possibility of suddenly acute events.

Further selected quotes relating to emotional demands are presented in Appendix B Table A4 (stressors: WT-E1-3; resources WTE4-9).

#### 3.1.2. Responsibility

Three SP, five CP, and one SN see stressors due to responsibility problems. More specifically, they mentioned to see stressors when the physician and patient could not communicate properly due to language problems (three CP and one SP), or when they had to educate patients who were not legally competent (one CP), making it difficult to educate patients properly.

‘A patient who is called for a clarification discussion but who comes with a caregiver, cannot be clarified in essential points’.

(CP 1)

Additionally, they reported that their employees were potentially burdened due to medical responsibility itself during complex procedures. (one SP), or in general because of a high sense of responsibility towards patients and colleagues that could lead to an overload for them (three CP and one SP). It was mentioned that first independent decisions in emergency services (one CP) could cause stress for young employees. Furthermore, managers reported to see stressors due to ethical dilemma when it comes to medical decisions (one CP and one SN), or unclear responsibilities due to the rotation system (one CP). The managers did not mention resources related to responsibility. Further selected quotes see Appendix B Table A4 (stressors: WT-R1-3).

#### 3.1.3. Decision Latitude

Decision latitude was the most often named resource after social support. Managers reported that their employees had the possibility to participate in the treatment or to decide completely on their own, as well as the possibility to further educate themselves in their field of interest. In addition, the employees have the opportunity to shape the working environment, participate in the duty planning, and to have a say in the recruitment of new colleagues (two CP, two SP, and two SN).

‘For example, the employees can already participate in the work scheduling and simply enter the yes, the needed free times or of course vacation’.

(SN 22)

‘I would never hire anyone either, so everybody has a say in that. I would never hire someone where the assistants say, no, it doesn’t fit at all, then we do not hire this person’.

(CP 15)

On the other hand, it was mentioned, that mostly also as a result of staff shortage, that decision latitude was restricted. For example, employees had to take over duties in other departments, even when they had their day off (two SN). In addition, it was described that the employees no longer had to take over the emergency service only in their own specialist area, but across all disciplines, which also might be a result of staff shortage in some departments (two CP).

‘When I have called everybody and they all say, “I don’t have time”, then I get the order that one has to come, even though he said he would not come. So, I sort of sign someone up for duty on their day off’.

(SN 18)

The obligation of detailed documentation was also perceived as a limitation of decision latitude, as the employees would then have less time for the actual task on the patient (three CP and two SN). Further selected quotes see Appendix B Table A4 (stressors: WT-DL1-2; resources WT-DL3-9).

#### 3.1.4. Qualification

With regard to the work characteristic qualification, different statements were also made, which seem to be related to the staffing. In case of too little personnel or time, a good induction of new employees seems to be difficult and some employees have to take over tasks for which they are not qualified enough or overqualified in order to fill personnel shortfalls (one SP, two CP, and four SN).

‘The employees have not had as good or sufficient time for familiarization. They are practically thrown in at the deep end, so to speak’.

(CP 6)

It was also reported, that a lot of the nursing staff had not learned to document correctly and were therefore overstrained, or that there were only few opportunities for advancement or further qualification for nurses, or that physicians had to take over tasks for which they were overqualified (e.g., documentation; one CP).

On the other hand, with sufficient resources, satisfaction was reported among employees when they were given tasks that matched their abilities (one CP), or when they were able to learn by spending time together at the patients through practical exchange of experience (two CP).

‘they are guided very closely and patients are cared for together with them’.

(CP 10)

The possibility of regular continuing education was also mentioned by one SP as a supporting factor. Further selected quotes see Appendix B Table A4 (stressors: WT-Q1; resources: WT-Q2-4).

#### 3.1.5. Information Offer

Regarding information offer, managers showed once more divergent perception. Four CP reported stressors due to the information offer that physicians had to chase after information, got wrong information, or that they got too much information from multiple sources, or that they even did not get important information early enough. Selected quotes see Appendix B Table A4 (WT-IO-1).

‘For example, you do not know whether a patient is being cared for or not. Whether a patient is ambulatory or not.”…” Nevertheless, it is regularly the case that we run after information’.

(CP 1)

Thus, while some felt that too much information was offered through too many channels, others found it very helpful, especially the clinic’s internal e-mail system and regular meetings (two CP and one SN). Another selected quote see Appendix B Table A4 (resource: WT-IO1).

‘The communication channel is also regulated by the distribution via e-mail account. Everyone has an e-mail account; everyone is obliged to check his e-mails on a daily basis. So that also the information, if the employees are there, then also flow. ”…” On the other hand, there is also further training, which we always do. We have meetings in the morning, where we discuss these topics’.

(CP 2)

#### 3.1.6. Variability/Diversity of the Task

With regard to the nursing staff, these stressors were mentioned with a focus on too great variability or diversity of tasks (four SN). It was mentioned that by less specialist work due to area care (nurses take over various tasks for less patients), versus function care (nurses only perform a few specific tasks on several patients), employees had to be trained in all kinds of specialist areas. Further selected quotes see Appendix B Table A4 (WT-V-1).

‘And now, simply because the employee key has become less, it happens that everyone has to do and has be able to do everything. And some people still have difficulties with things. That is, that they still have problems implementing what is demanded of them’.

(SN 23)

Contrary, one senior physician also reported, that the wide spectrum of treatment opportunity enhanced the task variability, and one SN said that they tried to relieve the burden of stressful tasks by changing them regularly.

‘I believe that the spectrum of what we offer here in our department—medical, internal medicine, cardiology—is very, very wide’.

(SP 33)

Another selected quote see Appendix B Table A4 (resource: WT-V1).

#### 3.1.7. Completeness of the Task

One SN reported to see stressors due to the completeness of the task, as there is no sufficient time to finish tasks completely.

‘We have no rest. That’s what I said before, no rest, as I said, in order to completely carry out a thing from A to Z according to a pattern. You have to be flexible all the time. As I said before, that is the thing that, as I said before, puts such a strain on us’.

(SN 24)

We could not identify resources due to the completeness of the task.

### 3.2. Managers’ Perspective on the Influence of Work Organization on the Employees

Stressors related to the work organization were mentioned most frequently by managers. In particular, stressors relating to the work process were reported most often, followed by stressors relating to working hours, and stressors relating to communication or cooperation were reported least frequently (Table A2). Resources relating to work organization were mentioned as the third most common, particularly resources relating to communication or cooperation were mentioned more often compared to the work process and working hours (Table A3).

#### 3.2.1. Work Process

We were able to find several factors that influence the work processes according to the managers. In terms of content, we found that especially staff shortage (four CP, ten SN, and four SP) along with vacancies that cannot be filled (two CP) was most commonly named as a factor that influences work processes by all groups.

‘The greatest burden, I would really say, the constant understaffing’.

(SN 26)

Additionally, other stressors related to working process can be seen as a consequence of staff shortages, such as high work density (five CP, three SP, and five SN) or heavy workload (six CP three SP, and eight SN) amongst others due to documentation obligation (one SN),

‘Workload, that sometimes you cannot meet the patients’ needs due to lack of personnel’

(SN 17)

‘I guess one of the major burdens is just the amount of work’.

(CP 13)

‘The intensive workload, be it intensive in the sense of the frequency of what needs to be done. Patient care, writing physician’s letters’.

(SP 36)

(Constant) time-pressure (three CP, one SP, and six SN),

‘Enormous time pressure, among other things’.

(CP 6)

‘The time. So due to the drastic staff reductions’.

(SN 18)

Coordination problems, because not all task fields can be operated at the same time (one CP),

‘Staff shortage which leads to some coordination problems, because not all task fields can be operated at the same time’.

(CP 5)

Or processing of several tasks simultaneously (one SN and two SP).

‘It was so stressful, the chief of staff, the attending physician, they both talk, one from the left, one from the right, you have to say: ‘Hold on, I can only do one thing at once’.

(SN 21)

**Figure 3:**
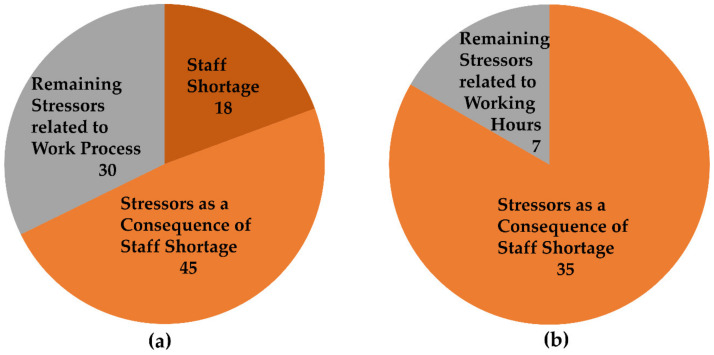
Graphical representation of (**a**) stressors related to the work process divided into the subcategory staff shortage, stressors as a consequence of staff shortage, and remaining stressors related to the work process and (**b**) stressors related to working hours divided into the subcategory stressors as a consequence of staff shortage and remaining stressors related to working hours. The distributions are based on the count in Table A2. The numbers indicate the number of mentioned work characteristics per defined group and across all occupational groups.

A comparison of the statements regarding the subcategories shows that staff shortage and its consequences account for a considerable proportion of stressors related to work processes (Figure 3a). Thereby, some interviews revealed that in some areas, staff shortages affect the nursing sector even more than the medical sector.

Next to staff shortage, managers reported to see impaired work processes for their employees due to inconsistent patient care and little continuity in the work (two CP, one SP, and one SN), high patient fluctuation (one SN and four SP), or because they must perform at call in emergency situations and thus have to be continuously available (one CP, two SN, and one SP). Furthermore, factors impairing work processes were work interruptions (one SN) due to patients knocking at the door (one SN) or constant ringing of patients (one SN), and in general, unclear work processes due to changes in structures (three CP and two SN), waiting for colleagues in team meetings leading to longer working hours (one CP),

‘To do a quick team meeting, but at that time the other work is not yet done. For example, five or three of them are in the operating room and one is still running somewhere to do something. Under these circumstances you cannot manage to start the meeting at the scheduled time when you have acknowledged that’.

(CP 5)

Or regarding discharge management, when the letters of the physicians were not yet ready (one SN), no clear time schedule for new admissions, discharges, or interviews with relatives (one SN) accompanied by poor bed management (one CP), or unfavorable distribution of work between departments (one SP), and no rotation plan, which leads to loosing employees to other departments (one SP).

On the other hand, one SN reported to have changed the work process to protect the employees from having a too close a relationship with the patients,

‘At the moment we are accustomed to weekly changes with the children. So in the past we took care of a patient from admission to the end, if possible with the same nurses, which was quite good for the parents, they had fixed contact persons, but that was not so favorable for the staff, so we said we would rotate more, so that we don’t build up such a strong bond. An example, we do not always succeed in that.’.

(SN 17)

Or due to the new legislation that a higher personnel key unburdens the employees (one SN). One SN reported relief through handovers directly at the patient’s bed instead of separately in the duty room, or that the employees are relieved by additional assistance personnel (one SN).

‘This means that the patient arrives, registers directly with the ward secretary and the ward secretary works and then continues to give the instructions, the patient has come. So, this is already well received. Just like with the nursing auxiliary, who then does this ward help. If you know that someone got you covered, I don’t have to wipe all the tables and such, because someone will come and do it. This is definitely a relief, I can now invest five more minutes for the patient’.

(SN 23)

Further selected quotes see Appendix B Table A5 (stressors: WO-WP1-12; resources: WO-WP13-14).

#### 3.2.2. Working Hours

Similar to work processes, a closer look at the stressors related to working hours shows that a large part can be attributed to the factor lack of personnel such as too many overtime hours (six CP, one SP, and five SN),

‘Overtime hours are necessary to compensate for the follow-up service. This means that the employee usually stays one or two hours longer. I already have 88 overtimes hours this year, the other employees have about 60 to 70.’.

(SN 20)

High number of emergency services (five CP and two SP),

‘So, I have to, let’s say the month has 30 days on average, so with this number of employees I have to do at least six services or seven services. And this really consumes the employees over the years, so to speak.’

(CP 6)

Little or no break time (one CP, three SP, and two SN), or that there are no common break times possible (one SP, one SN).

A comparison of the statements regarding the subcategories shows that stressors of working hours as a consequence of staff shortage account for a considerable proportion of stressors related to working hours (Figure 3b). Only the remaining stressors regarding working hours like shift work at weekends and on public holidays (one SP and one SN), family incompatible and exhausting shift work (one CP, one SP, and two SN), exhausting night shifts (two CP and one SN), unfavorably arranged shift work (two SP), which leads to too little recovery time between shifts (two SN), especially when employees must be taken off vacation (two SN), can be seen as factors that cannot be attributed to staff shortages but are part of hospital work in patient care.

‘Because the shift and night services are also exhausting. We always have people around, mothers with children who cannot cope with shift work’.

(CP 9)

Only one SN reported to see some positive aspect regarding working hours by trying to maintain regular breaks. Further selected quotes see Appendix B Table A5 (stressors: WO-WH1-2).

‘We try to implement certain aspects like 11 o’clock coffee, that you really take a short break in between and then collect yourself before you continue, so we try to keep these things going.’

(SN 18)

#### 3.2.3. Communication/Cooperation

With respect to communication and cooperation between the departments, the managers replied ambiguously. While eleven managers reported poor communication and cooperation between departments, leading to further disruptions in the workflow, such as tasks that cannot be completed because information from other departments is missing (five CP and four SN), which also affects discharge management (one SN), or includes that too little important information is documented in the internal documentation system (one SN),

‘This group is so unorganized, so disorganized. This one is so big that one hand doesn’t know what the other is doing. You regularly become desperate here’.

(CP 3)

‘We notice again and again that patients are badly prepared, that one does not keep to agreements.” …” If a patient is to be called up and he should be able to walk, but he is lying in bed, then the question arises: how is that possible?” … “If someone makes an order and the order is not obeyed, then you just have to ask yourself what is going on’.

(CP 1)

Four managers (one CP, two SP, and one SN) were very positive about cooperation with other departments. There seems to be a difference in the nature of the cooperation. Especially regarding formal matters, such as the transfer of information, or insufficient work in advance, there does not seem to be good cooperation between departments. Cooperation through personal help, such as the loan of personnel in case of staff shortages, or supportive interventions after stressful events seems to be possible through good agreements.

‘We have already foreseen this situation. And we have been exchanging personnel over and over again in recent years, precisely so that we can support each other’.

(SN 22)

With regard to the relationship between physicians and nursing staff, the results were also divergent. While some managers mentioned tensions between the professions, partly due to differences in staffing (one CP and two SN),

‘if you do this together with the physicians, because they are often, I think, not even aware of what consequences are caused by a tiny little action of them, if they are simply half an hour late for a joint visit, for example’.

(SN 18)

Others reported good cooperation and agreements between the two professions (one SN).

‘So, we have a pretty good relationship with our doctors, we also get along with them relatively well. We work hand in hand a lot. We don’t have to chase after them. They’re almost always there’.

(SN 18)

It appears to be related to the management style, as good cooperation between the two professions was expressed with flat hierarchies in particular, and to the time available for joint meetings.

With regard to internal cooperation, there was the criticism that responsible persons are often difficult to reach, but that on the other hand the ethics committee can greatly relieve the burden in dilemma situations. There were similarly different judgments for the area of cooperation with external collaborators. While cooperation with facilities for aftercare, transport, or social services for patients seems difficult (one CP, one SN, and one SP),

‘If the bed manager is not there, there is no substitute and then everyone has to try to get along somehow. It’s relatively simple’.

(CP 15)

Other managers are grateful for the possibility of external supervision.

‘Where physician and patient sometimes are in difficult situations even an ethics committee, which can also sometimes help us in exceptional situations’.

(CP 9)

Another cluster of stressors related to poor communication is, i.e., too few opportunities to talk about difficulties within the departments or problems with patients on an interdisciplinary basis (two SP) or in general because of no open discussion culture (one CP and two SP), and poor communication between the hierarchical levels (one CP) or even conflicts between different medical faculties (one SN). It was also stated, that they spent too little time together on the patient, inter alia due to too much time for documentation (one SP), or, that there does not exist a rotation plan with other departments (one SP).

‘…we have no rotation plan, no fixed one, so we have also lost two employees to exactly such departments’.

(SP 35)

Additionally, poor availability of responsible persons (one CP) was another factor that stresses employees in situations when decisions have to be made that cannot be made by themselves.

‘…tries to reach the nursing service, she does not reach anyone, writes an e-mail, no reply. Maybe after three hours you’ll get an answer, see how you get along’.

(CP 15)

Further selected quotes see Appendix B Table A5 (stressors: WO-C1-4; resources WO-C5-12).

### 3.3. Managers’ Perspective on the Influence of Social Factors on the Employees

Most managers reported trying to compensate for the demanding work with social support. In fact, social support from managers was the most frequently mentioned resource (see Table A3 and Figure 2). Stressors due to social factors were named third most frequently by managers. Stressors due to colleagues were mentioned more often as compared to stressors due to managers themselves (Table A2). On the opposite, resources related to social factors were mentioned most often, especially resources through support from the managers themselves (Table A3).

#### 3.3.1. Managers 

Regarding support by the managers, they reported that they tried to find out about the needs of their employees through active conversation offerings (five CP and seven SN) such as annual employee appraisals (four CP and two SN), and by generating an open atmosphere (five CP, one SN, and one SP).

‘In my position, 12 h a day is largely due to the fact that in the evening, when everyone is away, everyone knows the boss is there and I can just go in and talk to him. And that’s just the way it is. That’s important. Important for each individual. For me, of course, it adds up with many employees’.

(CP 7)

‘I also have annual employee appraisals and when I notice that the mood is changing somewhere, I get all the people together at one table and try to moderate things’.

(SN 17)

In stressful situations or in the event of employee illness, it was reported that the managers tried a motivating communication style (one CP and two SN), and expressing a supportive, back-strengthening attitude (one CP and one SP).

‘That it’s just totally motivating when you give people the confidence to do something and say, now you decide, it’s okay, I’m behind you, but you do it. And that’s right. And that you might be able to break through people’s reserve with something like that, people who might be a bit more cautious. And then also always with that, always with the background that they know it’s all supported by the attending, by the chief resident’.

(SP 38)

In general managers stated that they tried to strengthen the team climate, amongst others by trying to be a role model (two SN), to handle errors or overtime hours in a constructive way (two CP, one SN, and two SP),

‘We work hard on the processes to improve them, to create a stable team, to train employees further’.

(CP 10)

‘Another point is, it is also very important to send people home, for example, when there is not much to do. Some don’t dare to do that here. They all have enough overtime. And sometimes the three of them sit in the ward at three o’clock in the afternoon, there’s nothing more to do, and I have to say that I directly address them and say which of you will go home now, because it doesn’t make sense. And I also think that it is important that people who are staying longer should be allowed to do so, it is actually only fair’.

(SP 38)

To make the duty roster fair (two CP, three SN, and one SP),

‘*From my point of view as a ward manager, try to make good duty scheduling*’.

(SN 25)

To distribute tasks fairly (two SN), or to unburden the employees by moving them from stressful tasks to less stressful areas (one SN).

‘So, if the personnel key allows and then we sometimes deduct them from the very intensive’.

(SN 17)

They even step in when there is a shortage of staff and leave their own tasks (four SN, two SP,

‘So that I often say, I’ll do it, I’ll do it, I’ll do it, so that this person when I realize he’s on the edge’.

(SP 38)

Try to get more staff to unburden their employees, i.e., by contacting the managing director (three SN),

‘Because then I just said, ‘Enough is enough. With this staffing, I can no longer provide the patients with the care that is necessary for them. And at that point, people really reacted to it’.

(ID 27)

Additionally, to try to meet the supervisor’s duty of care in case of illness (one SN).

‘When I notice that the person concerned is not doing so well physically, they will of course talk to him. So, it is then always appealed to the instinct of self-preservation. So, sometimes, you must really show them, make them think, think a little bit more about yourself, if you were to call in sick right now, we could get help. But if you come, we won’t get any help. And whoever is here has to perform at 100, 110, 120 percent. Coming to work sick is more harmful for the team than not coming at all.’.

(SN 26)

Additionally, contact initiation from the managing director, which showed the employees that the manager tries to support them, was stated as supportive (further statements see Table A6 SF-M34-51).

‘And only recently the managing director has repeatedly managed to find a few words that simply yes, give a little bit of support. That is not much, but nevertheless, it shows that your effort is noticed, in fact’.

(SN 22)

On the other hand, some managers said that they saw stressors for their employees due to an unsupportive attitude, e.g., that some had no or little perception of psychological stress among employees (one SN and two SP), or that they looked away (one SP) and did not care of about needs of the department (one CP), or that there was lack of support by responsible persons (i.e., upper manager and works council; one CP and two SN), e.g., team gets no supervision, or that promises from upper management were not kept (one CP). Two managers said that they observed a lack of appreciation (one CP and one SN).

‘I think we can more or less agree on the fact that health, the staff’s health, does not have such a high priority. Because if you thought about it, you wouldn’t call people to work on their days off, you wouldn’t let them work 200 h overtime, because at some point you have to realize that in the long run that’s not healthy for some people.” … ‘Somehow there is a lack of sensitivity when it comes to determining who is able to work under how what kind of pressure’.

(SN 18)

Furthermore, managers stated seeing stressors because of the behavior of managers themselves such as little constructive communication in general (one SP), no constructive dealing with criticism and mistakes among managers (one SP), yelling of managers at staff under a time pressure due to acute events (one SP), claims, and impatience towards the employees (one SP).

‘Yes, proper handling is very important. I also experience this time and again in a stressful situation, for example, that I am very demanding. That I also do not tolerate delays because I am fighting for a human life’.

(SP 37)

Two managers said that they observed a lack of appreciation (one CP and one SN).

‘Yes, and otherwise I must honestly say that we have actually been waiting for some sort of praise by the management or nursing service management for years. This hasn’t happened in years. So those simple words, you did a great job, yes, something like that, yes, or something, it has never happened’.

(SN 22)

#### 3.3.2. Colleagues

With respect to work characteristics related to colleagues, we could again find opposing statements from the managers. While some of the managers observed a good team spirit (four CP, two SP, and four SN)

‘*We have despite all these issues we have a good atmosphere*’.

(CP 15)

‘*I think we have all together always tried very hard to form a group that excludes no one. Each according to his abilities, some people are better at this, others at that, but there is no scapegoat*’.

(SP 34)

Additionally, they stated that employees carried out joint activities with the team outside working hours (four SN), or talked about problems as a team and had a good exchange within the team (three SN and two SP), others reported difficulties in team building (two CP, two SP, and one SN), little team spirit especially between physicians and nursing staff (one SN), and interpersonal conflicts in the team (two CP), often because of personal sensitivities, miscommunication, and lack of critical faculties (two SN).

‘The people, people are not really used to criticism either. The fact that there are always higher demands, you have to discuss many things that could be improved and people often take this too personally immediately, which is then of course not helpful for the whole team spirit and the team’s strength, I say this now.’.

(SN 17)

Similarly, managers reported that employees supported each other (two CP and one SN), especially in situations with a staff shortage to the point that they sacrificed themselves for each other (one SN), e.g., by taking over services from vacation, although they themselves still needed rest and regeneration,

‘Because you can really tell that they are at the end of their rope, to put it in plain English. And yet they somehow manage to trudge through here. Some of them, where other colleagues would call in sick for minor issues, really crutch their heads under their arms to work here, because the team as such is actually already strengthened. And nobody wants to bail on the other in that moment. So, there is definitely some kind of attitude or motivation of not wanting to let the others down behind it.’.

(SN 26)

Additionally, on the other hand, others assessed self-sacrifice, inter alia to compensate for the decreasing working abilities of older employees (one SN), as potentially harmful to health (one CP), especially when, specifically among physicians, it is devalued as not being able to work under pressure (one CP).

‘Overload is often also seen as a weakness and therefore not mentioned by the employees’.

(CP 13)

Additionally, as a result of heavy workload lacking assumption of responsibility was reported, i.e., that work is left for the next one, as one only works on the patient for a short time (one SP) or so called “management-by-waiting” (one SN), which means doing nothing at first in the hope that it will resolve itself on its own or because of colleagues.

‘This high workload cannot lead to a good team building. And I believe that this team structure is also an important criterion in medicine, because you always act as a department.’

(SP 35)

‘Management by waiting, for example. I just sit it out. Somebody else will takes care of it.’

(SN 19)

Further selected quotes see Appendix B Table A6 (stressors: SF-C1-4; resources SF-C5-21).

### 3.4. Managers’ Perspective on the Influence of the Work Environment on the Employees

Regarding work environment managers named rather less factors and it seems to be related to the building where the managers worked whether they reported positive or negative factors. This is, because one building of the hospital was still in the old part, while the other building was already in a new part. Hence as stressors relating to the work environment, too few (one CP and one SP) or too small rooms (one SN), a twisty building and too long distances (one SN), too few or too small waiting areas (one SN), an unclean on-call room (one CP) or dirty hallways (one CP), not enough computers for all physicians (one SP), or a failure-prone medical device were mentioned (one SN), along with difficulties in dealing with new technology for older employees (one SN).

‘There are not enough computers here for all doctors and not enough rooms for everyone.’

(SP 38)

‘It is just when we are sitting there with three people during the rounds, plus the two doctors, then this room is simply too small for so many voices.’

(SN 21)

‘For example, we also lack premises where patients can wait sensibly. This means that some of them are really piling up in the corridor’.

(SN 26)

On the other hand, two managers (one SP and one CP) said that they saw good technical equipment leading to satisfaction among their employees.

‘The working conditions here in the unit are very acceptable for us, because we have the complete technical equipment here to immediately recognize acute deterioration or serious clinical pictures or to recognize and treat them relatively quickly. This means we are provided with the necessary equipment here in order to create good working conditions.’

(SP 37)

‘So, what is motivating is that we now have a whole range of new devices that allow us to expand our therapeutic and diagnostic spectrum and which also improve workflows.’

(CP 15)

With respect to ergonomic factors, one nurse stated stressors due to stressful physical work during mobilization and basic care.

‘These constant admissions, discharges, recordings. The physical is the one thing we have in basic care, the mobilization and this pressure, to get the patient healthy as quickly as possible so that he can be discharged.’

(SN 24)

### 3.5. Managers’ Perspective on the Influence of New Working Conditions on the Employees

We did not find any expressed stressors or resources that fell under new working conditions, such as spatial mobility. Although we identified stressors regarding reduced delimitation between work and privacy, we placed them in the category of work organization. This is, because we saw this as a result of a lack of staff and difficulties in staffing the services. On the other hand, we were able to identify further stressors and resources from the interviews, which did not fit into the GDA system and are described in the following section.

### 3.6. Other Factors Influencing the Employees’ Mental Health

Next to the above-mentioned work characteristics, the managers perceived further potential stressors or resources. For example, we found reduced resilience of older workers (one SP and one SN), or that stress from the work task does not match the personality profile of the employee (one CP), or additional stress situations from private life (one CP) that increases stress perception at work.

‘There are competent doctors who are not able to handle certain situations well due to their character or it is just not so easy for them, others handle it very well, or at least seem to do so.’

(CP 13)

Similarly, it was reported that the elimination of primary nursing, which means the assignment of one nurse to a certain group of people in need of care, leads to anonymity and dissatisfaction, since less appreciation can be experienced and the employees feel interchangeable (one SP), or that highly professionalized, well-structured working according to algorithms, guidelines, and evidence means that there is no possibility of involving personal informal exchange in between (one SP). Finally, many changes due to restructuring measures (one SP) were mentioned as an additional stressor. Additionally, denial of emotional stress or exhaustion contributes to the fact that stressful situations cannot be dealt with constructively (one SP).

‘Highly professionalized, well-structured sector working according to algorithms, guidelines and evidence. This means that there is no possibility of involving your psyche in between.’

(SP 37)

‘Nobody talks about how bad a day was, nobody says they will probably not make it tomorrow. People also try not to admit to themselves that they are burdened emotionally. And I think that’s something that naturally leads to people always carrying their emotional tension with them.’

(SP 37)

We also identified resources that did not fit into the GDA-category system. One SP stated that he found it very supportive that the hospital made offers that had nothing to do with medical training in the hospital, such as sports courses,

‘So, there are also various athletic activities here. But also, and this plays a very important role for me, these are medically inactive stories’

(SP 31)

or good strategies to deal with stress within a department (one SP),

‘Yes, all the way to oncology, where one, which is really also care-intensive and one experiences how, my impression in this area is that they have a very good strategy for dealing with the stress.’

(SP 36)

or that employees can give overload indications (two SN) or an attempt is being made to obtain personnel on loan (one SN).

‘Overload indicators can be made. But there it is about the burden of too much work, if you are only two people instead of three on a nursing ward or something, or if you are alone and have severe cases, you can’t cope with, then you can do that.’

(SN 21)

‘Yes, and if we can no longer help ourselves alone, we turn to nursing service. And, of course, hiring temporary staff is the last resort.’

(SN 22)

## 4. Discussion

This qualitative interview study aimed to investigate which of the known stressors and resources that are relevant to employees’ health are perceived by hospital senior managers in their area of responsibility and what importance they attribute to them. By analyzing thirty-seven interviews with fourteen CP, nine SP, and fourteen SN, we could identify a large number and large variety of stressors and resources, which we assigned to the category system according to the GDA guidelines.

Overall, senior managers reported more stressors compared to resources. Concerning stressors, the most frequently mentioned stressors related were to work organization, followed by works task, social factors, and work environment. On the other hand, the most frequently reported resources were related to social factors followed by work task, work organization, and work environment. Altogether, stressors relating to work organization were by far the most frequently named, followed by resources relating to social factors, and stressors relating to the work task.

Interestingly, we could observe a large number of contradictory statements between the managers. In terms of communication and cooperation between departments, we received ambiguous results. In formal matters, such as the transfer of information, cooperation between departments seems to be difficult. Cooperation through personal assistance, such as hiring staff in case of staff shortages, seems to be perceived as very supportive. With regard to the relationship between doctors and nurses, the results were equally divergent, which seems to be related to the management style, as good cooperation between the two professions was reflected in flat hierarchies in particular. Similarly, in relation to the work task, an ambiguity was found in the work characteristic emotional demand. A large positive part of the hospital work seems to be nourished by the experience of good treatment and satisfied patients, and at the same time, dealing with serious illness and death of patients and the grief of their relatives was reported to be one of the greatest emotional burdens. Regarding decision latitude stressors were mentioned with a focus on the need to make decisions between a great variability or diversity of tasks, and on the other hand, a wide spectrum of treatment opportunity was stated as being attractive for the employees. This result fits with the observations of Kubicek, Korunka [31], who found that a low and higher level of decision latitude lead to higher levels of irritation and was associated with higher burnout levels in the long term. Ambiguous statements were also made concerning qualification, as in case of too less personnel or time, a good induction of new employees seems to be difficult, and, on the other hand, if resources were sufficient, satisfaction among employees was reported when they were given tasks adapted to their level of performance. Relating to information offered, some felt that too much information was offered through too many channels, others found it very helpful, especially the clinic’s internal e-mail system. Most managers reported that they tried to compensate for the demanding work with social support, and on the other hand, some managers indicated that they saw stressors for their employees due to an unsupportive attitude, e.g., that some had little or no perception of psychological stress among employees or that they looked away and did not care about the needs of the department. With regard to work characteristics in relation to colleagues, we were again able to find contradictory statements from managers. While some of the managers observed a good team spirit, others reported difficulties in team building, little team spirit, and interpersonal conflicts within the team.

In summary, depending on the general circumstances, managers consider certain work characteristics to be more supportive or more health threatening. The results fit in with the current debate on work design research, which suggests that attention should be paid to specific configurations of working conditions [40]. Very often a lack of personnel was reported as a moderating and mediating factor. For this reason, we will discuss the connection between staff shortages and other stressors in the next section.

### 4.1. Staff Shortage as a Core Problem

In analyzing the interviews, we came across the fact that especially staff shortage was the most commonly named stressor by all groups (eighteen out of the thirty-seven interviewed participants). We also recognized that other stressors related to working process were seen as a consequence of staff shortages, and similarly, a closer look at the stressors related to working hours shows that a large part can be attributed to the factor lack of personnel. Furthermore, we saw that some of the stressors mentioned above, such as having to endure patients suffering because there is no time to take care of them all at the same time, are due to a lack of personnel. It was also mentioned that due to a staff shortage, employees had to take over tasks in other departments, which they did not feel up to. In addition, it was reported that due to a staff shortage there was hardly any time for a good initial training so that many employees felt overstrained at the beginning. It was found that the structured and efficient working method leaves little time to talk about other issues such as mental stress, which makes the situation even worse.

**Figure 4:**
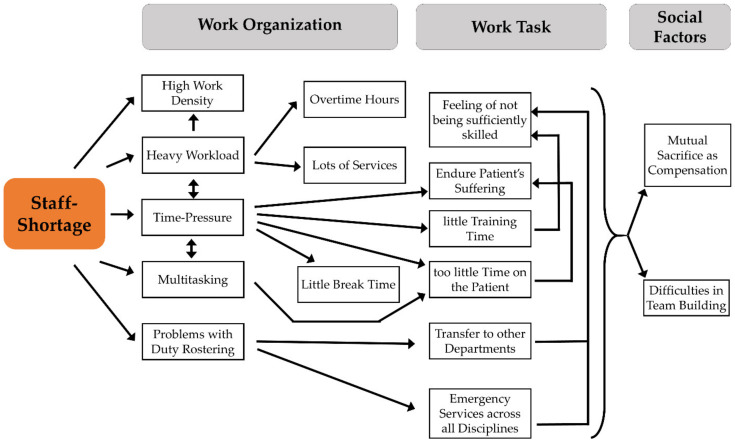
Graphical representation of hospital managers perceptions of the effect of staff shortage on further stressors relating to work organization, work task, and social factors according to the hospital managers.

Summing up, we could identify that hospital managers perceive staff shortage as an underlying core problem for a variety of other potentially hazardous work characteristics relating to work organization, work task, and social factors (Figure 4). According to our impression, the managers mostly try a lot in their power to reduce the burden on the employees, in particular through their personal support. Nevertheless, it seemed to us that even a major effort on the part of the managers could only counteract the overwhelming number of other stressors with difficulty.

Regarding a lack of professionals and staffing problems in hospitals, Blum [41] found on the basis of basic data of German hospitals [42] that in 2016 approximately 60% of all hospitals had staffing problems. About every second hospital could not fill vacancies in the nursing service. Compared to 2011, the problem of filling vacancies has also increased significantly. In contrast, the problem of filling physicians’ positions has decreased minimally from 2011 to 2016 but remains a major problem [41]. A meta-analysis of 35 studies investigating the effect of nurse staffing levels on patient outcomes found, which higher staffing levels were among others associated with lower in hospital mortality, less medication errors, and less infections [43]. We therefore propose that an improvement of the personnel situation in hospitals is a major underlying problem that would have to be reduced by measures. Blum [41] suggests that this problem can be reduced by measures such as the expansion of training capacities and the reduction of part-time employees by extending the working hours of part-time workers. In addition, important steps may be the improvement of working conditions through occupational health management, the introduction of age-appropriate working models, or the relief of employees from documentation and administrative tasks.

### 4.2. Results in the Light of Relevant Stress Models

A meta-analysis of 37 studies found that especially three main conditions (imbalanced job design, occupational uncertainty, and lack of value and respect) in the occupational context are associated with the development of common mental disorders [44]. Within these main conditions, further factors were identified that were associated with greater risk of developing common mental health problems: high job demands, low job control, high effort–reward imbalance, low relational justice, low procedural justice, role stress, bullying, and low social support.

The results of the interviews allow a reference to these common stress models: the job-control model [29] focuses on certain aspects of the job profile that allows or denies employees the experience of autonomy and self-efficacy. According to this model, those persons who are exposed to high quantitative requirements (e.g., permanent time pressure) without having sufficient control and decision-making power over the execution of their work (decision latitude) are particularly at risk of health problems due to work stress. According to this, it is not the quantity of work per se that endangers health, but the performance under conditions of low controllability of the work process and work content. We found that employees in hospitals work under permanent time-pressure and have to react to unforeseeable events, so that most of the staff is externally determined in the execution. Moreover, in the results of the interviews, we found that in particular the transfer of the employees to other departments due to a staff shortage leads to reduced decision-making power. However, there were also indications that managers perceived too much decision-making as a burden for employees see Kubicek, Korunka [31]. Importantly, the extension of the model by the component “social support at work” postulates that work stress is aggravated by the fact that the burdens resulting from a lack of autonomy and self-efficacy, and from the lack of stimulating experiences and learning opportunities at work, are not mitigated by helpful social support from colleagues and superiors [45]. In considering the difficulty of, for example, familiarizing new colleagues with the company and supporting them due to a staff shortage or time, it can be assumed that this factor is even more of an aggravating factor. In line with this, Jalilian, Shouroki [46] showed that in nurses high mental and physical work demands and low social support and decision-making scope was related to general fatigue and less job activity see also [47]. Similarly, Theorell, Hammarström [15] found a 74% increased risk of illness when working conditions are characterized by high demands and little control. On the other hand, Büssing and Glaser [48] showed that extended scope of activity is associated with increased job satisfaction, lower emotional exhaustion, and reduced complaints. An EU survey showed that 68 percent of employees complain about time pressure as stress and 79 percent about back pain or muscle aches. Interestingly, it was found that their primary causes are not ergonomic design deficiencies, but rather a loss of autonomy experienced in the work process [49].

Some manager stated that employees did not get positive feedback or positive appreciation about the course of a disease due to the job rotation system, or that appreciation from the managing director was lacking. Following the effort–reward imbalance model, which states that if a high level of expenditure is not matched by an appropriate reward, then “gratification-critical” stress reactions are triggered [28]. For employees, who are exposed to effort-reward imbalance at work, a meta-analysis of eight studies found a 49% increased risk of developing depression [50].

The model of organizational justice from organizational and social psychology focuses on subjective perceptions of unfair procedures in organizations [30,51]. According to this, a situation is perceived as unfair if the balance between expenses and earnings of the person concerned is less favorable than that of a corresponding reference person. Kivimäki, Vahtera [52] showed that experiencing injustice is accompanied with a doubling of the risk of developing depression. With respect to our results from the manager’s point of view, employees are treated fairly and equally with regard to roster distribution and allocation of tasks, dealing with errors.

Therefore, despite managers’ attempts to treat employees fairly, in view of our results and the findings to date on the above-mentioned stress models, the situation potentially poses a health risk for hospital employees.

### 4.3. Results in the Light of the Factor Social Support and The Psychosocial Safety Climate

Three effects are attributed to social support processes: 1. they are able to reduce the level of work-related stress, 2. have a positive function in coping with stress (i.e., the stress is perceived as less demanding), and 3. have a health-promoting effect, because health-promoting resources are built up as a result [53,54]. Social support by the managers and among colleagues was the most frequently cited resource. With regard to collegial social support, it could be shown that the effect of mutual social support, which is particularly evident in collegiate meetings, has a positive effect on resources, including the decision-making scope [55].

However, the effect of social support strongly depends on the psychosocial safety climate. The psychosocial safety climate (PSC) concept, developed in Australia, captures the priorities and readiness of an organization to protect the mental health of its employees. It is assumed that the PSC within work organizations predicts work conditions and in turn psychological health and engagement [56]. It has been found that the PSC is largely driven by senior management, and it is related to job demands, psychological health problems, and emotional exhaustion outcomes [57]. According to our interviews, the managers’ perception of the burden placed on employees and their willingness to support them could certainly be assumed. It could therefore be considered that this contributes to a good PSC, which has been shown to strengthen mental health [56].

Havermans, Boot [58] found that a lower psychosocial safety climate score was associated with significantly higher stress in employees, and that autonomy and social support measures diminished the relation between psychosocial safety climate and stress by 12%. Hence, although future research has yet to confirm the results, it can be assumed that social support can only to a small extent act as a buffer in a poor PSC. It therefore seems more relevant to look at the PSC than at the level of social support alone. In addition, a recent study suggested that medical and nursing managers in hospitals themselves need to get more work-design competencies and decision latitude to get more control and to be able to sufficiently counteract stress factors [59]. We therefore propose for future research to investigate how the PSC influences the perception of the managers.

### 4.4. Limitations of the Study

Due to the voluntary participation of managers in the interview study, it can be assumed that we primarily reached those managers who already had a positive attitude towards the topic of employee mental health. We cannot therefore rule out a certain sampling bias. Furthermore, it cannot be ruled out that the participants in the interview situation showed socially desirable response behavior. The result that social support by managers was seen as the most important resource could be interpreted against this background. Furthermore, we only interviewed the managers of one hospital, so that a generalization of the results may be limited. However, the interview guideline appears to be suitable for use in other hospitals, so that the results could be verified in further studies. Additionally, since we examined the managers’ perspective on the psychological burdens of their employees, we could not make any statements about the actual burdens on employees. Future research could, for example, use mixed methods to investigate the actual psychological well-being of employees and the corresponding work characteristics using questionnaires or parallel interviews or focus groups.

## 5. Conclusions

The result shows that the managers’ perspectives on health-promoting and health-threatening work characteristics concerning their staff largely coincide with those of accepted work stress theories. The most significant stressors were reported from the field of work organization. Staff shortages in particular were identified as an underlying stressor for a number of other stressors relating to work processes, work task, or social factors. Especially regarding the work task, we also found task-inherent stressors, specifically with regard to emotional demands. Stressors relating to the work environment seemed to be of secondary importance. Social support by managers or among colleagues was mentioned as the most common resource. According to the interviews, managers strive to reduce the burden on their staff, especially through their personal support. Nevertheless, it seemed to us that even a great effort on the part of managers could only counteract the overpowering number of other stress factors with difficulty.

## Figures and Tables

**Table 1:** Demographic description of the sample.

	Chief Physicians	Senior Physicians	Senior Nurses	Total
female	2	2	9	13
male	12	7	5	24
total	14	9	14	37
Age range (years)	43–60	38–60	34–60	34–60
Mean age (years)	51.9	43.6	47.9	47.8
Mean number of years employed	5.4	8.7	23.4	12.5
Departments	Anesthesia, dermatology, gynecology, vascular surgery, cardiology/intensive care medicine, pediatrics and juvenile medicine, hospital hygiene, hand and plastic surgery, pneumology and sleep medicine, radiology, spinal surgery, vascular surgery, psychiatry, urology, and internal medicine.	Anesthesia, cardiology, neurology, pneumology and sleep medicine, spinal surgery, urology, and hand and plastic surgery.	Oncology and hematology, pediatric and youth intensive medicine, anesthesia, occupancy management, sleep laboratory, internal intensive medicine, trauma surgery, general surgery, pediatrics and youth medicine, spinal surgery, geriatrics, and psychiatry.	

## Appendix A

**Table A1:** Category system for interview analysis.

Characteristics Area	Sub-Categories	Possible Critical Expression
**Work Task**	Emotional demands	by experiencing emotionally very touching events (e.g., dealing with serious illness, accidents, death) by constantly responding to the needs of other people (e.g., customers, patients, students) through permanent showing of demanded emotions independent of own sensations threat of violence from other people (e.g., customers, patients)
	Responsibilities	unclear competences and responsibilities
	Decision latitude	The employee(s) has/have no influence on work content workload working methods/-procedures sequence of activities
	Qualification	activities do not correspond to the qualification of employees (over/underchallenge) insufficient briefing/introduction to the job
	Information Offer	too extensive (stimulus satiation) too low (long periods without new information) unfavorably presented information Inadequate (important information missing)
	Variability/Diversity of tasks	one-sided requirements: few, similar work objects and work equipment frequent repetition of similar actions in short cycles
	Completeness of the task	Working activity involves: - only preparatory or - only executive or - controlling actions only
**Work Organization**	Working hours	changing or long working hours unfavorable shift work, frequent night work extensive overtime inadequate break regime work on call
	Work process	time pressure/high work intensity frequent faults/interruptions high clock rate
	Communication/Cooperation	isolated single workstation no or little possibility of support by superiors or colleagues no clearly defined areas of responsibility
**Social Factors**	Colleagues	too low/too high number of social contacts frequent disputes and conflicts Nature of conflicts: social pressure situations lack of social support
	Managers	no qualification of the managers lack of feedback, lack of appreciation lack of leadership, lack of support in case of need
**Work Environment/Equipment**	Design of the Workplace and of Information Stimuli	unfavorable working areas, spatial narrowness inadequate design of signals and notices
	Work Equipment	missing or unsuitable tool or work equipment unfavorable operation or setup of machines insufficient software design
	Physical and Chemical Factors	Noise Lighting Hazardous substances
	Ergonomic Factors	unfavorable ergonomic design hard physical work
**New Working Conditions**	These characteristics are not the subject of supervisory action but play a role in the stress situation of employees.	spatial or temporal flexibility reduced delimitation between work and privacy

**Table A2:** Number of mentioned stressors per manager und subcategory.

Main Categories	Sub-Categories	Chief Physicians	Senior Physicians	Senior Nurses	Total
**Work Task**	Emotional Demands	11	6	12	29
	Responsibility	10	3	1	14
	Decision Latitude	5	-	4	9
	Qualification	2	1	4	7
	Information Offer	4	-	-	4
	Variability/Diversity of tasks	-	-	4	4
	Completeness of the Task	-	-	1	1
	**Total Work Task**	32	10	26	68
**Work Organization**	Work Process	30	21	42	93
	Working Hours	15	11	16	42
	Communication/Cooperation	14	7	12	33
	**Total Work Organization**	59	39	70	168
**Social Factors**	Colleagues	6	3	6	15
	Managers	4	7	4	15
	**Total Social Factors**	10	10	10	30
**Work Environment**	Workplace	3	1	4	8
	Work Equipment	-	1	2	3
	Ergonomic factors	-	-	1	1
	Physical and chemical Factors	-	-	-	-
	**Total Work Environment**	3	2	7	12
**New Working Conditions**	-	-	-	-	-
**Other Stressors**	-	2	5	1	8
				Total	286

**Table A3:** Number of mentioned resources per manager und subcategory.

Main Categories	Sub-Categories	Chief Physicians	Senior Physicians	Senior Nurses	Total
**Work Task**	Emotional Demands	1	2	4	7
	Responsibility	-	-	-	-
	Decision Latitude	4	2	4	10
	Qualification	3	1	-	4
	Information Offer	2	-	1	3
	Variability/Diversity of tasks	-	1	1	2
	Completeness of the Task	-	-	-	-
	**Total Work Task**	10	6	10	26
**Work Organization**	Work process	-	-	4	4
	Working hours	-	-	1	1
	Communication/Cooperation	3	2	6	11
	**Total Work Organization**	3	2	11	16
**Social Factors**	Colleagues	6	4	13	23
	Managers	32	7	31	70
	**Total Social Factors**	38	11	44	93
**Work Environment**	Workplace	-	-	-	-
	Work Equipment	1	1	-	2
	Ergonomic Factors	-	-	-	-
	Physical and Chemical Factors	-	-	-	-
	**Total work environment**	1	1	-	2
**New Working Conditions**		-	-	-	-
**Other Resources**		-	2	3	5
				Total	142

## Appendix B

**Table A4:** Selected quotes of the participants related to the work task.

**Emotional Demands**
WT-E1: The delivery of bad news. For example, a prognosis or even brain death, or in case of serious diseases that lead to death over a very long period of time. (SP 36)
WT-E2: I work in a specialty where the clinical pictures are already very difficult, and the problem is that we have patients, that is not Mr. Müller, Mr. Meier, Mr. Schmidt, it IS Mr. Müller, we know him, we have accompanied him for years. And we usually care for them until they die, and then it’s not just a patient who has died, but Mr. Müller who has died. (SN 16)
WT-E3: Staff have to respond immediately to changing events. There’s always a certain basic tension in the process, you can’t get rid of. (SP 37)
WT-E4: The surgical successes that we have, that after an operation, the patients are not only satisfied with the operation itself but also with the way we treat them and that to a large extent. (SP 32)
WT-E5: here lies no one with a cold, confronted with the need to preserve life. That usually works out pretty well. We have a high success rate. (SP 37)
WT-E6: Especially with the seriously ill patients, or even less ill patients, it is already a good feeling when you can help. (CP 13)
WT-E7: So, you actually see motivation with the trauma surgeons, for example, they usually come after accidents or so, when they can walk again or the gait training begins in general and they are then perhaps transferred to geriatrics for rehabilitation, that is of course already a motivation, I always think when patients get to walk again. (SN 23)
WT-E8: There are motivating experiences indeed. So of course, I was just talking about the babies, when they have grown nicely, when they are doing well, when the parents go home happily, this is transferred to the employees, who are also happy. Or when we had very seriously ill children, who then go home after a certain time, we are also happy. (SN 25)
WT-E9: Great patients, you like to work with, where you can see successes who go home well. That is simply beautiful. Really still nice. Fascinating, exciting, new. (SN 29)
**Responsibility**
WT-R1: Especially in dealing with patients with whom communication is difficult. In other words, we have many patients here in this hospital, of course, which is simply due to the situation, with a migrant background, who often cannot speak our language. And it is a burden for the staff to be able to adequately communicate with the patients. (SP 32)
WT-R2: In addition, of course, the medical responsibility itself during complex procedures. (SP 31)
WT-R3: Situations where we partly find ourselves in an ethical dilemma when it comes to medical decisions. (CP 9)
**Decision latitude**
WT-DL1: A burden that we have because of the so-called administrative activities. And that which is nerve-racking in everyday now speaking of me personally, this is something we have observed with colleagues. Because when things get stuck, so to speak, you get the feeling that you can no longer really take care of the actual job you have learned. (CP11)
WT-DL2: Then you have to figure out what to leave out. First and foremost, according to priorities, the documentation that is not necessary or that at least for the moment does not seem necessary. The supply of medication is important. (SN 20)
WT-DL3: The opportunity to design areas responsibly and to have the resources to do so. To recognize and define for yourself what is important for patients, to be there for them, in an adequate way. And what you need for that. And then also in fact have the opportunity to be able to shape this. (CP 7)
WT-DL4: That I also give him the opportunity to deepen his own experiences in special examination techniques. And he also has the opportunity to develop and shape this area. … ‘that you have the opportunity to organize yourself and shape your working environment (SP 36)
WT-DL5: That it’s just totally motivating when you give people the confidence to do something and say, now you decide, it’s okay, I’m behind you, but you do it. And that’s alright. (SP 38)
WT-DL6: They work relatively independently, they know all the activities, they can actually do what I do, from the evaluation and so on. Some employees work independently and say I’m going to finish this and tell me that. (SN 21)
WT-DL7: So as long as the shop is running and we stick to hygienic guidelines, we are allowed to do and act as we want. (SN 27)
WT-DL8: It is often the case that we then agree on doing certain operations more often or being allowed to learn more or developing further or supervising doctoral students, answering scientific questions, something like that. (CP 12)
WT-DL9: Hey, have you ever thought of having people write their own rosters?’ I’ll just try that out. Of course, it’s always difficult with three shifts, but I try to make it possible, things like that, so that you say: ‘Okay, we can try it. If everyone notes down which shifts are most suitable for them, we’ll just see how it works, if that works.’ I definitely try to avoid such service obligations. And I always try to find out if something can be done or made fit differently, so that nobody needs to come additionally. (SN 18)
**Qualification**
WT-Q1: As I see it, jobs in the care sector are relatively ungrateful because there are too few opportunities for advancement and development, and that is a problem. (SP 36)
WT-Q2: But the satisfaction, I would say, I see at least also with my employees, if they can successfully complete tasks, depending on their level of performance, of course they can and want to be entrusted with new tasks. … I also believe that it is definitely pleasant for employees, even for a younger assistant physician in further training, when they made the right decision there, when he came to the right diagnosis in emergency service. (CP 9)
WT-Q3: I think I also really strengthen them when it comes to conveying specialist knowledge, and then supporting them, demanding and encouraging their way of thinking in an evidence-based way. (CP 3)
WT-Q4: that I also give the opportunity to deepen his experiences in special examination techniques.” … “then I think it’s essentially the case with assistants that you teach them something in terms of training, that they learn something new. In return, I also expect that patients are properly cared for, that the documentation is correct, that the physician’s letters are correct. And then on our part, the opportunity to learn physical neurological examination and perhaps also to learn one or the other part, or an intervention, which is relevant for the internists. (SP 36)
**Information offer**
WT-IO1: So, we also have handovers in the morning, which used to be exclusively medical. Now everyone is invited. So, care, therapists, social workers. So that we all meet at half past eight in the morning for about half an hour and then we also discuss what was going on in the services, what is to be discussed organizationally, which transfer is scheduled. (SN 29)
**Variability/Diversity of tasks**
WT-V1: So, if the personnel key allows and then we do the following, they are then deducted from the very intensive and then first make a week of rearing the children or something like that to come down again then. (SN 17)

**Table A5:** Selected quotes of the participants related to work organization.

**Work Process**
WO-WP1: The situation is different if only one person out of the three positions is appointed, or two people are appointed. Then it does not work that way. Then this person has to work or think at two positions at the same time or handle the tasks one after another. (CP 5)
WO-WP2: At the moment when several patients are causing problems, it’s like you’ve read before, that you are actually just running past it and noticing: ‘Oh, he has just pulled his artery, I should actually go in there, but I can’t, because you have to go back there. And I think that is the most stressful thing for people, that you can’t work with foresight anymore, but you’re really just working, you’re just reacting to what’s happening right now. (SN 18)
WO-WP3: We’ve got a lot of work, it’s really stressful. Of course, it is also difficult for some people because of the work density. (CP 15)
WO-WP4: In the past, there were handover books where you wrote a sentence to a patient, finished. Things are much different today. It is yes, everything has increased. And the staff has decreased. And everything else has increased. (SN 25)
WO-WP5: In the field of anesthesia, the time pressure in the operating room is certainly a stress factor for employees. (SP 31)
WO-WP6: …Another problem is, this inconsistency of work, because services exist, so this is a problem that has also arisen with the Working Time Act, that I now have more shift systems and colleagues go to services or other functions and therefore constant patient care is no longer possible. In other words, they look after a patient for a day and then go somewhere else again. And then they eventually see him again. This causes a lot of dissatisfaction in my work and also in the work of my colleagues. (SP 36)
WO-WP7: Appointment compression simply by short retention periods, relatively high turnover, you have to adapt to new patients relatively quickly again and again. Has relatively little time for the individual patient. Simply the high flow of patients. (SP 40)
WO-WP8: …that the pressure is not even. We have days when the ward can be full to the brim and I can comfortably sit in the kitchen with three others and drink coffee and then I have days when I only have four patients and I don’t know how I am going to look after these patients with three colleagues. There are really high load peaks, this can happen all of a sudden. There is no pattern or regularity. I cannot assume that this will always be the case in winter or in summer or on Thursday or on Friday, or whatever. There is no regularity. It can happen any second, relatively quick. (SN 18)
WO-WP9: It’s mentally exhausting when the phone is ringing all day long, I remember that from my old employer, there I also sometimes sat with the secretary next door and she also did the bed planning and then the phone went from morning to evening and then still in the evening, she was worn-out from all these phone calls, all this writing down, the things she had to keep in mind, you must not forget anything, you have to keep track of everything, she was harassed by the phoning and by writing down, by noticing, you mustn’t forget anything, you have to keep track of everything. (CP 12)
WO-WP10: In many areas there is no clear definition of the employee’s task. Due to frequent rotations, people are confused and do not know. And then they are sometimes, this is extremely frustrating when you don’t know what I have to do, what I am responsible for, what you are not responsible for. (CP 13)
WO-WP11: But what I also partly deal with is the current discharge management, where doctors can’t get involved, for whatever reason, patients are often discharged as late as in the afternoon because the letters are not ready yet. (SN 20)
WO-WP12: …but we have no rotation plan, no fixed one, so we have also lost two employees exactly to such departments. (SP 35)
WO-WP13: Now that we have new legislation, that we have to have a higher personnel key on level one in the intensive care unit in order to guarantee one-to-one care, that helps us to the extent that when we have reached this personnel cut at some point, we simply have more time for the whole thing and not always have to chase work, which is often the case. (SN 17)
WO-WP14: Delivery at the bedside.… Our colleagues have seen many advantages of that. As I said, the fact that this first round of the late shift, which is already superfluous, because there are two colleagues, the early shift and the late shift, who take care of the patient together, this means that certain things that the early shift might not have noticed are immediately recognized by the late shift. (SN 24)
**Working Hours**
WO-WH1: When a patient has died, he is prepared, brought down and the work continues immediately. There is not even a moment where you can take some time to reflect and say, well, okay, that’s done now, and I want to go and have a sip of coffee and then continue. Sometimes that’s missing. (SN 23)
WO-WH2: …that is, length of service, rotating shifts every weekend. In my opinion, there are not enough buffers. (SN 19)
**Communication/Cooperation**
WO-C1: or even the pick-up and delivery service simply does not come and does not pick up the patients and so on. (SN 18)
WO-C2: So, the choir spirit, is not as you might want it to be. This is due to the great organizational complexities of this house. There is not one large group, but many small ones, which often change among themselves. (CP 13)
WO-C3: In my everyday life I experience that there is far too little direct communication with each other, instead a lot of communication takes place in writing via entries and consults, because you do not spend enough time together with the patient. So, I think that we do not spend enough time together in joint visits. Nursing, assistant physician rarely visit together, where problems could be discussed directly together. (SP 36)
WO-C4: When things get stuck, so to speak, you have the feeling that you can’t really take care of your actual job, could you have learned, because you are too busy, making appointments, asking questions, organizing things that could actually be delegated, but often, these things cannot properly be implemented so you end up doing all of it yourself. CP 11)
WO-C5: Do not want to say that it is not, but then to call, I am not feeling well today, I will not come to work today. And yes, I’m on duty tomorrow, but I’ll come to duty then and do the whole thing. So, there is so much awareness, sense of responsibility, because otherwise they know very well that there is a gap, someone else has to fill. (CP 6)
WO-C6: Specifically, that of course we have additional staff available, who then take over such overlapping functions that we always have specialists available, SP available, who help people in such critical medical situations and provide assistance. (SP 31)
WO-C7: We have a lethality conference which takes place once a week, where such topics are also discussed. (SP 32)
WO-C8: For example, a colleague of the chief physician told me that she had had a particularly stressful situation in the intensive care unit with relatives. And what kind of supportive offer we have in our department for her employees as support, where we could come and talk, a supportive offer for her employees as support. Yes. We do that on the spur of the moment. Short-term, informal interventions, as needed, yes. (CP 4)
WO-C9: Which is fine, I don’t know if you mean that, we have a psychooncologist in the house. That is quite good, because whenever I really notice that there is a need for a conversation or there is a physician with a new disease pattern or a new condition and unfortunately we cannot take the time to sit down and talk to him, then we switch her on and she has the time, takes the time and then we have the feeling that we have done something for the patient anyway. (SN 16)
WO-C10: we also have a nursing case meeting. There we can also address certain issues or problems, but this is for the whole house. (SN 16)
WO-C11: but we do have supervision on our side. So, and this is also clarified within the framework of supervision. (SN 29)

**Table A6:** Selected quotes of the participants related to social factors.

**Colleagues**
SF-C1: So, there’s this tension like, where do I see myself on the team, how do I deal with my colleagues. How do I fit in with the team. (CP 9)SF-C2: So, the choir spirit, it is not as you might want it to be. (CP 13)
SF-C3: The team as such just wants to get out even more, more, more. And thereby compensating for structural weaknesses to the point of self-sacrifice. (CP 7)
SF-C4: Physical aches and pains come with increasing age, or rather no longer aches and pains, but real problems and the whole team suffers from them. So those who are sick and those who are still there have to cope with it and then they also get sick at some point. I wish I had a mixture of young and old. (SN 16)
SF-C5: And I have a good team with a good atmosphere, we also exchange ideas with each other. So that works out well. (SN 25)
SF-C6: The interdisciplinary cohesion, but also in our department the very close cooperation, where contact and conversation is possible at any time. (SP 32)
SF-C7: We work extremely well together as a team, that has to be said. That’s also, you get along with the people, that’s also real, there are no people, almost without exception, that you would not consider as part of the team here. (CP 2)
SF-C8: To have a team together that thinks and feels similarly so to say. And that knows each other so well that you know the personal strengths and weaknesses in the team and of course you can compensate for them. (CP 7)
SF-C9: Most of the employees I know are motivated in their job and I think that is very, very important for the team spirit. (CP 5)
SF-C10: Team spirit is pretty good I’d say. (SN 17)
SF-C11: that we’re also having dinner with the team. That we not only meet at work and talk about patients and stuff. But they go out, and we go out to dinner. (SN 16)
SF-C12: So, on the one hand, I think we motivate each other ourselves. That we not only meet at work, but also privately from time to time. And then we have a barbecue or whatever. (SN 22)
SF-C13: what is quite important for us is that we have a relatively good team, we have grown together really well, they have known each other for a long time and they like to work together, some of them also spend time together apart from work. I think that makes a big difference. (SN 18)
SF-C14: I say, for private time, that we do this together. For example, we were planning a trip. (SN 28)
SF-C15: That’s why it’s also important that we do a lot of let’s call it mental hygiene with our team, also colleagues among each other, I’d say. (SN 16)
SF-C16: So, we often talk about what patients trigger and cause in us. We always call this our little group. This is certainly connected to the field of expertise, our colleague is still a psychiatrist, so in this respect we are more familiar with perceiving what patients trigger in us, what situations also trigger in us. And occasionally reflect on this together during the lunch break. (SP 36)
SF-C17: We also laugh a lot, we also fool around, and we sometimes talk about private things, that is also completely legitimate.” … “the one who would intercept this emotional crisis of the other. Usually it is the best colleague or the one who is currently on duty with whom you get along well. That you talk about it. (SP 37)
SF-C18: Otherwise we have regular station meetings and if such a case gets out of character, supervision. (SN 17)
SF-C19: The cooperation among the employees, the feeling that they support each other. (CP 4)
SF-C20: So, we are definitely trying to help each other. (SN 24)
SF-C21: Sometimes it also happens that the group itself then says “okay, can I take something off your hands?” and that would certainly be another point to strengthen that. (CP 13)
**Managers**
SF-M1: Employees also do not get the feeling that they receive the support they need, because he knows exactly that six positions are missing. (CP 6)
SF-M2: It was planned and agreed by me that she would then remain as a specialist, in her three-quarters position, so that it would be compatible with family life, for an unlimited period, so that she could, she had this promise. When this examination came, I also passed this on to her, suddenly, different than what had been agreed upon beforehand, the statement that she could only be employed for a limited period of time. This might seem like a small issue from afar, but for such a person it is dramatic. Dramatic for her, dramatic for me too, because it contradicts my promise. (CP 7)
SF-M3: That they know they have a boss who stands up for them. I think I’m doing pretty well. At least, that’s what I hope anyway. At least that’s the feedback I get. It’s very important to me. So, the duty of care for the employees. (CP 15)
SF-M4: A certain closeness means addressability for me. My employees know that they can actually address me at any time. (CP 5)
SF-M5: Or the comforter when a colleague’s brother has recently been diagnosed with cancer. That ranges from business situations, where supervision is required, to very pragmatic situations, in the sense of an open ear in family stories. (CP 4)
SF-M6: If someone here has any topic family, partner, work, employee, colleague, that the door is open at any time, that they can relieve themselves here. And every time someone says he has to go to physiotherapy at 3 pm, I would never stop him. So that’s when they get—and I’ve said this quite often—my full support immediately. (CP 3)
SF-M7: Seeking direct conversation. Try to... yes, like I said, try to have a direct conversation first, to find out if this is right... sometimes I hear about it from others. And there you can only accompany or give support. (CP 1)
SF-M8: Is important, I think, because an open communication structure, so yes, you hear it again and again in the management seminars, the open door of the boss, but that is also lived here. (CP 2)
SF-M9: I am also very close to the staff. We really support them a lot, they can come to us with any questions. (CP 10)
SF-M10: But I would say that I have an open mind to look at that as well. And where you then notice that employees are perhaps, let’s say, harder on themselves, you can also follow up. That would now be the second thing, the intervention. So that you basically not only perceive that, but basically also look, okay, can I then relieve someone, simply take them out of a situation. (CP 11)
SF-M11: Through team meetings and regular get-togethers, eating together, for example, we already try to create an atmosphere in which you can get rid of non-work related issues or by having a certain proximity to the employees, “…”So we have relatively flat hierarchies, I also listen to it when I notice that one—this does not only concern the physicians it also concerns nursing and the other therapists, who are located somewhere between the nursing and the doctors in their work and who are also very stressed. If I notice this, I invite them to a conversation as well. (CP 13)
SF-M12: Of course, we also try to discuss such things when they are dramatic things that also happen here in everyday work. (CP 9)
SF-M13: So, they also come to me, even if it has nothing to do with the profession as such, and they also come to me with very private questions, problems and worries. (SP 33)
SF-M14: we try to arrange it that way when the employees come, I always say: ‘come anytime, whenever there is something wrong.’ (SN 17)
SF-M15: We brought it up there and tried to talk about it. What could be done differently, that you don’t get the feeling, what have I done wrong. There are definitely a few possibilities. (SN 16)
SF-M16: To have conversations and try to really reach people. (SN 26)
SF-M17: When I see that someone is suffering, conversation is the way to go, a very brief five-minute conversation, or “Is there anything I can do here? Is something wrong? You just ducked out?” Sometimes it’s also private things that are included in the job. For example, when your father has passed away. And the employee is then completely shocked on duty and I don’t know what to do in the first place. You have to see if you can get someone from somewhere else and say, “Go home.” Overtime cutbacks, or even the legal cutbacks that you have when a parent dies. That’s important. (SN 20)
SF-M18: in my ward, I definitely pay attention when I see that someone is depressed, or maybe I in a way notice that they are somehow different or something, I talk to them about it and always ask them if something happened, if something is going on. (SN 23)
SF-M19: I can read faces. If I see that you are not well, I can easily recognize that, then I take him aside and ask him what worries and troubles him, but never in front of the others, rather in a four-eye conversation. (SN 25)
SF-M20: So, we do team meetings. (SN 28)
SF-M21: So, I don’t know how often I sit here with staff and really take them aside, even after work, just talk to each other for ten minutes after the handover, just to listen, check the situation, how are you doing right now. What can I do for you, because I notice that you are under pressure? What do you need right now to feel a little bit relieved? I’m trying to do whatever is possible for me. But especially as their superior, there are also some things I cannot do. (SN 27)
SF-M22: You just have to be careful not to disturb, trying to defensively prevent escalation and you can only do this to a certain extent if you are not like: That is your fault, you have to go through this now, but if you say: “look, I still have three or four emergencies and I can still help you” or “leave it alone, you’d better do it”. Provide clear structures to make employees feel a sense of relief. (SN 20)
SF-M23: Communication with each other. This is something I tried to convey relatively early, that you have to treat everyone friendly and respectfully. (CP 1)
SF-M24: Something I really like is highlighting the things that have been going well in the last weeks and months. Which of course is also a bit motivating for the team. (SN 26)
SF-M25: But I think we have found this way here, which should actually make it relatively easy for the people to feel comfortable, I think. (CP 12)
SF-M26: Try to create a certain atmosphere. (CP 13)
SF-M27: Well, I’ve been in charge of the ward for two years now and I’ve introduced the idea that we also have dinner with the team. (SN 16)
SF-M28: So, I have to say, I am a calm person that means it doesn’t matter how much stress there is or how much we are burning I try, I am in rage, but I try to stay calm. Because when I am excited, then my employees do the same and then everything goes wrong so you have to stay calm and get an overview, and I hope I succeed in that. (SN 16)
SF-M29: I also know that it’s good for me to stay calm and then say, “Do this, do that, do that.” I’ve also heard that from colleagues, they say, “How can you stay so calm under all this stress?” “Yes,” I say, “On the outside, I’m calm. It doesn’t help when I’m nervous here. (SN 21)
SF-M30: Go home early, send... if possible. (CP 2)
SF-M31: The other is perhaps measures where you try to create temporary help in the workloads, so that you can create more space for recreational value, in an open space, so to speak. (CP 11)
SF-M32: But there is significantly more overtime in winter than in summer. But there is a good cooperation with the nursing management, which accepts this and says that overtime will be reduced in summer. (SN 25)
SF-M33: If you say I somehow need a week’s vacation, that I’ll say, look, how about taking a week’s vacation and then immediately afterwards a week off overtime, then I’ll try to cycle the vacation planning so that the employee doesn’t have to sit on like 300 overtime hours, but can also reduce them promptly. And that does not mean reducing them according to what is best for me or for the company, but also when it suits the employee, as well. (SP 35)
SF-M34: I think everyone I talk to about this, we always talk once a year, a further training talk and personal development talks. (CP 12)
SF-M35: We talk about personnel development with our employees at least once a year. This concerns the level of professional training, but also the level of personal development. (CP 9)
SF-M36: And we do these talks, these personnel development talks. They sometimes tell you what bothers them. (SN 28)
SF-M37: We conduct regular employee appraisals. (CP 10)
SF-M38: Employee appraisals, we do those too, of course. (CP 15)
SF-M39: Yes, you try, let me put it this way, to accommodate the employee by saying, okay, fine, you arrange certain leisure activities that fit into your system. (CP 6)
SF-M40: So, we have a very transparent vacation schedule, a very transparent duty roster, so that you can also see who has done how much work and who does what when, so that there are no great inequalities, so that it’s not the case that some people work the whole weekend, several weekends in a row, and others not at all. On the contrary, there is a way of ensuring that there are equal rights. (CP 2)
SF-M41: In the rostering, that you give everyone a goodie and then people jump in when you want them to. So that’s where I think it’s very important to keep everyone equal. (SN 17)
SF-M42: So, I try to equally distribute it, not always the same people, so to say, I say, when it is about coming for a weekend more, I do my best. (SN 18)
SF-M43: I try to fulfill the wishes of the employees as good as possible. For example, the employees can already participate in the duty planning and simply enter the yes, the desired services where they need free time or vacation anyway of course. And we try to respect that. (SN 22)
SF-M44: That one also looks at the distribution, that it is also fair. (SN 17)
SF-M45: I’m just trying to spread the load evenly, like I said. (SN 18)
SF-M46: I have an eye on the mental health of the employees, respond to their wishes regarding holiday planning, further education wishes, duty roster design, a support by taking over tasks of the employees. (SP 31)
SF-M47: So that’s when you try to remind people, ‘Take the break.’ Then I say, ‘Yes, I’m here, I’m doing this work. Now you go and leave. (SN 21)
SF-M48: When I’m on duty with a colleague and simply recognize her facial expression, certain yes, characteristics now, because she’s about to burst. Then I say, then I’ll go in between and say, I’ll take over certain things, I’ll take it from you. But okay, I can do that. (SN 24)
SF-M49: Sometimes there is no other way and we also try to relieve ourselves, so that not one of us is standing there all the time, especially when we have long reanimation or so that we always change, so that not only one of us is affected and we try to take the other patients away from the one who has a lot to do with his patients, so that he can just concentrate on one. (SN 18)
SF-M50: I try to reduce the burden by asking the nursing service management to hire more staff so that the few employees we have don’t have too much work, too much overtime, which shouldn’t happen at all. In that case I don’t know whether they do not want to do it or cannot do it, because they might have received instructions from the hospital to save on personnel or something. (SN 21)
SF-M51: I see it as my responsibility to look out for my employees, to check the workload. And do I perhaps have to offer them a helping hand and talk to my superior to check if the number of staff needs to be increased because the mental, psychological and physical strain is simply too high for the staff who are currently working there. (SN 23)
